# Bridging the gap between bariatric surgery and continuous multidisciplinary care

**DOI:** 10.51866/lte.514

**Published:** 2024-02-07

**Authors:** Mohamad 'Ariff Fahmi Ahmad Zawawi, Mohammad Shukri Jahit, Barakatun-Nisak Mohd Yusof

**Affiliations:** 1 MB BCh BAO, PGDip, MMed, Klinik Kesihatan Seksyen 19, Jalan Gelora 19/46, Shah Alam, Selangor, Malaysia. Email: ariff.fahmi@gmail.com; 2 MD, MMed, Fellowship in Upper, Gastro-Intestinal Surgery No. 4, Jalan P7, Presint 7, W.P. Putrajaya, Malaysia.; 3 BS Dietetics, PhD, Research Fellow in Diabetes Nutrition, Department of Dietetics, Faculty of Medicine and Health Sciences, Universiti Putra Malaysia, Serdang, Selangor, Malaysia.

**Keywords:** Bariatric surgery, Primary care, Obesity, Metabolic surgery, Dumping syndrome

**Dear editor,**

We read with great interest the case report by Cheah et al. on dumping syndrome in a woman who became pregnant shortly after bariatric surgery.^1^ The recently launched clinical practice guideline (CPG) has declared obesity as a complex and chronic disease with heterogeneous presentation.^2^ Thus, personalised and continuous care is essential for people living with obesity (PLwO) even after bariatric surgery.

From an upper gastro-intestinal surgeon’s perspective, dumping syndrome among patients undergoing bariatric surgery and gastrectomy for malignant or benign conditions rarely occurs nowadays owing to proper preparation and educational support. From the start of weight management programmes to the perioperative phase, vital information and counselling are provided. Further guidance is pertinent immediately after surgery to help patients eat and live after bariatric surgery. The abovementioned case report is an impetus to emphasise the importance of adequate preparation prior to bariatric surgery and the need for continuous multidisciplinary postoperative care. An establishment is needed to regulate the clinical standards of bariatric practices in Malaysia to ensure their integrity, safety and efficacy.

The increasing prevalence of obesity has seen a parallel rise in demands and supplies of bariatric procedures. The International Federation for the Surgery of Obesity and Metabolic Disorders (IFSO)’s Global Registry reported 100,092 bariatric surgeries performed in 2014 alone and an exponential rise to 394,431 surgeries performed in 2018.^3^ Further, bariatric surgery tourism services have also been booming. Azlan et al. analysed websites of a range of international bariatric centres. They uncovered significant gaps pertaining to accreditation and provision of multidisciplinary care as well as a lack of follow-up with primary care physicians. Arguably, these issues may lead to poor health outcomes following bariatric surgery.^4^ An analysis of 293 Facebook pages and 122 Twitter accounts revealed a large number of followers for commercial content related to bariatric or weight loss surgery. Conversely, the lack of followers for reliable educational support groups may have adverse impacts on patient lives and well-being.^5^

Whether bariatric surgery is perceived as a quick fix for obesity among the public is concerning. Moreover, healthcare professionals are prone to assuming bariatric surgery as a one-stop solution to ease the overwhelming complications of obesity. This tendency may result in inappropriate expectations and misconceptions that obstruct the provision of multidisciplinary and continuous care, especially after surgery.^6^ To fill this gap, primary care doctors should be equipped with bariatric knowledge and skills to conduct initial counselling, optimum assessment, prudent referral and continuous patient care.^7^

Bariatric and metabolic societies in South Korea, Japan and China have recommended surgical intervention at a body mass index (BMI) of ≥27.5 kg/m^2^ and ≥35 kg/m^2^ because Asians tend to develop diabetes and cardiovascular complications at a lower BMI.^8^ The American Society for Metabolic and Bariatric Surgery and the IFSO published a major upgrade that recommended bariatric surgery for Asians with a BMI of ≥27.5 kg/m^2^.^9^ Obesity CPG recommendations were based on the Malaysian Bariatric and Metabolic Working Committee’s consensus statement. Bariatric surgery is indicated for patients with a BMI of ≥32.5 kg/m^2^ and metabolic syndrome or cardiovascular risk and patients with a BMI of ≥37.5 kg/m^2^ without such comorbidities.^10^

Nevertheless, BMI alone may not accurately illustrate the impact of adiposity on cardiometabolic risk and quality of life.^7^ Considering the complexity and heterogeneity of BMI, there are evolving efforts to steer away from using this parameter in obesity risk stratification and therapeutic deliberation for PLwO, especially in the era of precision medicine. The American Association of Clinical Endocrinologists and American College of Endocrinology introduced adiposity-based chronic disease as a new diagnostic term.^11^ A group of renowned experts established the *Lancet Diabetes & Endocrinology* Commission on the Definition and Diagnosis of Clinical Obesity to identify criteria for its diagnosis.^12^ The American Medical Association has recently adopted a new policy to caution the limitation of BMI in clinical practice. Conversely, Edmonton’s Obesity Staging System and King’s Obesity Staging Criteria may facilitate prioritisation of patients with obesity and comorbidities for medical or surgical therapy.^13,14^

Relevant to the case report, we would like to emphasise the role of medical nutrition therapy (MNT) after bariatric surgery. Before discharge, patients should receive intensive MNT from a dietitian, focusing on gradual progression of dietary intake. Specifically to prevent and manage recurrent hypoglycaemia and dumping syndrome, patients are recommended to eat smaller and more frequent meals, up to six times per day, and consume fluids at least 30 min after each meal.^15^ Dietary protein is critical to support wound healing and protect against losing lean body mass. Patients should receive individualised prescription targeting 60–80 g of protein/day and up to 1.5 g/kg of adjusted body weight/day.^16^ Consumption of rapidly absorbable carbohydrates, especially food or beverages high in sugar, and alcohol should be avoided. Patients are also suggested to eat meals high in dietary fibre and lie down after meals for 30 min to delay stomach emptying. Long-term management should also involve preventing vitamin and mineral deficiencies, indicating that a proper pregnancy plan should be advocated to ensure optimal pregnancy outcomes.

A proper pregnancy plan is critical in the context of the case report: The foetus developed intrauterine growth restriction, and elective caesarean section had to be performed. Thus, effective contraception aiming for 12–24-month spacing, adequate macronutrient and micronutrient dietary intake and enteral supplementation or even parenteral nutrition in cases of hyperemesis gravidarum are crucial after bariatric surgery.^17^

Moving towards recognising obesity as a chronic disease, we urge administrators, clinicians and researchers in Malaysia to use first-person language such as PLwO in clinical practices and academic writings. A coalition of medical and public organisations, medical journals and academic institutions has released a joint consensus statement to end stigma in obesity. Weight bias and stigma can result in discrimination, undermining human rights, social rights and the health of afflicted individuals.^18,19^ This aspect is critical in primary care, where ensuring equitable access to holistic, multidisciplinary approach and continuous care is paramount.
